# The legacy of one hundred years of climate change for organic carbon stocks in global agricultural topsoils

**DOI:** 10.1038/s41598-023-34753-0

**Published:** 2023-05-09

**Authors:** Christopher Poeplau, Rene Dechow

**Affiliations:** Thünen Institute of Climate-Smart Agriculture, Bundesallee 68, Braunschweig, Germany

**Keywords:** Biogeochemistry, Carbon cycle

## Abstract

Soil organic carbon (SOC) of agricultural soils is observed to decline in many parts of the world. Understanding the reasons behind such losses is important for SOC accounting and formulating climate mitigation strategies. Disentangling the impact of last century’s climate change from effects of preceding land use, management changes and erosion is difficult and most likely impossible to address in observations outside of warming experiments. However, the record of last century’s climate change is available for every part of the globe, so the potential effect of climate change on SOC stocks can be modelled. In this study, an established and validated FAO framework was used to model global agricultural topsoil (0–30 cm) SOC stock dynamics from 1919 to 2018 as attributable to climate change. On average, global agricultural topsoils could have lost 2.5 ± 2.3 Mg C ha^−1^ (3.9 ± 5.4%) with constant net primary production (NPP) or 1.6 ± 3.4 Mg C ha^−1^ (2.5 ± 5.5%) when NPP was considered to be modified by temperature and precipitation. Regional variability could be explained by the complex patterns of changes in temperature and moisture, as well as initial SOC stocks. However, small average SOC losses have been an intrinsic and persistent feature of climate change in all climatic zones. This needs to be taken into consideration in reporting or accounting frameworks and halted in order to mitigate climate change and secure soil health.

## Introduction

Several lines of evidence suggest that climate warming is inducing a decrease in global soil organic carbon (SOC) stocks, mainly by accelerating soil microbial activity^1,2^. Despite huge uncertainties associated with the magnitude of SOC loss^3–5^, it is acknowledged as a powerful climate-carbon cycle feedback^6^. For this reason, modelling efforts on various spatial scales have been undertaken to estimate potential losses under different future warming scenarios^6–8^. It has been shown that SOC sequestration efforts for climate change mitigation, focusing on agricultural soils in particular, can be severely hampered by climate change per se^6,7,9^.

Several agricultural soil monitoring networks on a national to continental scale have reported SOC losses that are partly interpreted as being caused by climate change^10,11^. In one prominent case, the observed loss of SOC in agricultural soils in England and Wales has been attributed to climate change^10^. This interpretation was challenged in a subsequent study^12^, which found changes in management to be a more obvious cause of SOC depletion. This is likely to be the case in many parts of the world. Between 2009 and 2015, average losses in cropland SOC were also detected by the LUCAS soil inventory across the European Union and similarly in different member states of the European Union^11,13–15^. Despite a strong increase in yields and thus in biomass production when compared with pre-industrial agriculture, intensification may have caused declines in SOC due to agricultural management. For instance, high appropriation of net primary production, breeding to maximise C allocation to the harvested product, the application of pesticides, drainage, erosion, soil compaction and very frequent soil disturbances have most likely contributed to SOC depletion to varying extents^12,14,16–18^. Furthermore, land-use change can have long-lasting legacy effects on SOC trends^19^. It is thus likely that SOC stocks in many agricultural soils are not in equilibrium under current management practices^14^. Simultaneously, the global average temperature has risen since the beginning of the last century by approximately 1.07 °C^20^. Globally, the spatial variability of measured climate change is huge, with a tendency towards greater warming in higher latitudes. This reflects the difficulty of decoupling the effects of agricultural management from climate change-driven background losses. While efforts have been undertaken to quantify the total global soil carbon debt of land use^21^, no such assessment exists for past climate change.

In 2019, the Food and Agriculture Organization of the United Nations (FAO) published a map of global SOC stocks that was prepared with the participation of numerous countries^22^. Based on this map, the FAO subsequently created a framework to estimate the global carbon sequestration potential of agricultural soils^23^, which is high on the political agenda as a nature-based solution to combat global changes. This participatory framework (GSOCseq) used the well-known and widely applied SOC model RothC^24^ in combination with the simple and frequently used net primary production (NPP) model MIAMI^25^ to estimate how much carbon can additionally be stored in agricultural soils under certain simple scenarios of carbon input alterations. This pragmatic framework was established by leading experts in SOC modelling and can be readily applied in various contexts.

Here, the GSOCseq framework was used to derive the first spatially explicit estimate of past climate change effects on SOC stocks in global agricultural soils. Starting from today’s SOC baseline as derived from the FAO GSOC map, one century of climate data was used to backwards predict SOC trends for a total of 932 k points, equally distributed over the agricultural area in all climatic zones. Modelling was restricted to the area of applicability of the model framework, thus excluding organic, forest and urban soils. Two scenarios were modelled: in the first scenario, the steady state C input of 2018 was kept constant throughout the time series, so that only the climate change effect on SOC decomposition would be simulated (NPP_const_). In the second scenario (NPP_var_), the C input was scaled by the modelled NPP response to climate change (temperature and moisture only) to estimate how much of the SOC changes in NPP_const_ could have been compensated by increased biomass production. Those may serve as two different, context-specific baseline SOC trends of the past 100 years with a high spatial resolution.

## Results

**Figure 1:**
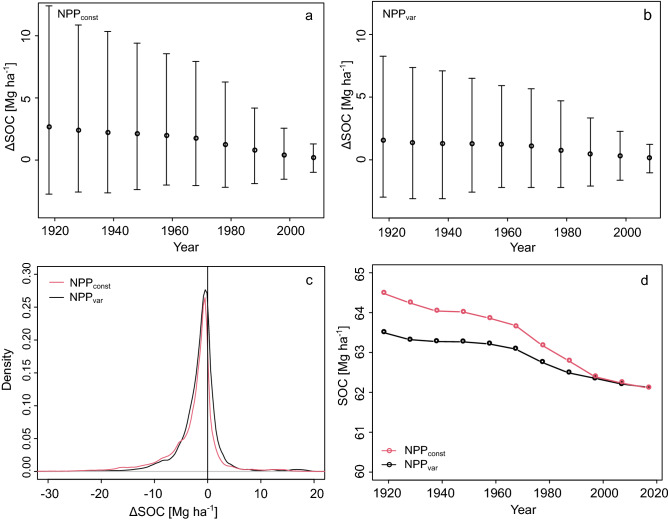
(**a**) Average climate change-driven SOC stock change since 1919 with standard deviations assuming constant C input (NPP_const_). (**b**) Average climate change-driven SOC stock change since 1919 with standard deviations assuming variable C input (NPP_var_). (**c**) Density distribution of SOC stock changes at all 932 k modelled points for both NPP scenarios. (**d**) Average global SOC stock of the past century at ten-year intervals as modelled from 2018 to 1919 with two C-input scenarios.

On average, global air temperature at the assessed data points increased by 1.03 °C between 1919 and 2018. Depending on the scenario, this has led to an average decline in total SOC stocks by 2.5 ± 2.3 Mg ha^−1^ (NPP_const_) and 1.6 ± 3.4 Mg ha^−1^ (NPP_var_) (Fig. 1a,b). The comparison of both scenarios shows that climate-driven changes in NPP (excluding CO_2_ fertilisation) were able to compensate for 36% of predicted SOC losses on average. For both scenarios, the majority of sites had losses in the range of 0–3 Mg ha^−1^ (Fig. 1c, 57% in NPP_const_ and 52% in NPP_var_). In line with the temperature trend, SOC losses were more pronounced in the latter half of the simulated timespan. A marked decline in average SOC stocks has been particularly evident since the late 1960s (Fig. 1d). Two different change rates were therefore calculated, i.e. for the first half and latter half of the timespan. This led to estimates of average climate change-driven SOC losses of 0.019 ± 0.045 (NPP_const_) and 0.010 ± 0.037 (NPP_var_) Mg ha^−1^ year^−1^ for 1919–1968 and 0.032 ± 0.059 and 0.019 ± 0.045 Mg ha^−1^ year^−1^ for 1969–2018 respectively.

**Figure 2:**
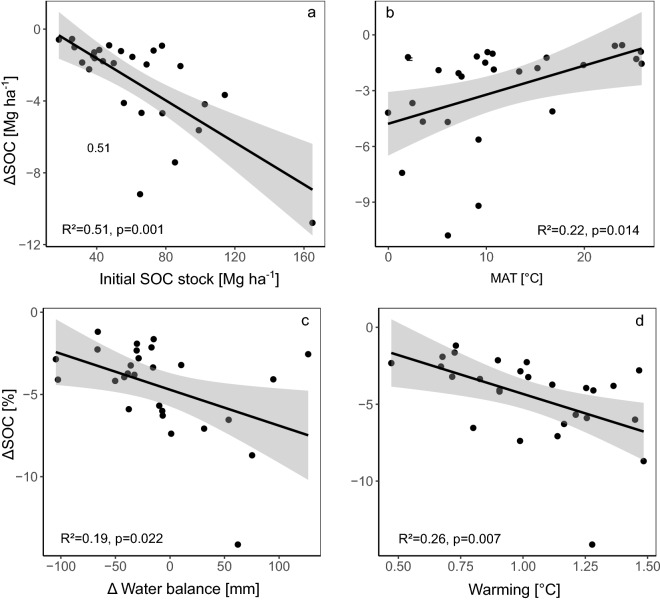
Average centennial (1919–2018) changes in soil organic carbon (SOC) stocks per climatic zone in the scenario with constant net primary production (NPP_const_) as a function of (**a**) initial SOC stock, (**b**) mean annual temperature (MAT), (**c**) changes in water balance and (**d**) degree of warming. Absolute (**a**,**b**) and relative (**c**,**d**) changes are shown.

SOC changes in response to warming also varied greatly between climatic zones (Table 1). Cold regions with high initial SOC stocks and the most pronounced increases in air temperature were predicted to have the highest SOC losses per area (Fig. 2a,b). However, also relative losses tended to be highest in colder regions (Table 2). Temperate ecoregions showed intermediate losses, and both tropical and arid regions tended to be the least affected (Tables 1, 2, Fig. S1). When NPP and thus biomass input was kept constant, on average SOC was lost from all climatic zones, ranging from − 10.8 ± 7.2 Mg C ha^−1^ in the temperate Cfc climate to − 0.6 ± 1.2 Mg C ha^−1^ in the hot arid BSh climate (Table 1). However, SOC losses were not only driven by the initial SOC stocks and mean annual temperature of the respective climate zone; relative SOC changes were also correlated to changes in water balance and temperature, i.e. higher losses were observed when the climate became wetter and warmer (Fig. 2c,d). Accordingly, the largest SOC losses in the NPP_const_ scenario could be expected where high initial SOC stocks, high increases in mean annual temperature (MAT) and mean annual precipitation (MAP) coincide (ΔSOC = 4.501–0.057 × SOC_initial_ − 4.318 × ΔMAT − 0.019 × ΔMAP, R^2^ = 0.74). When climate change-driven NPP alterations were considered, on average SOC was also lost from all climatic zones. In the NPP_var_ scenario, SOC changes in the past century ranged from − 6.4 ± 7.0 Mg C ha^−1^ in a cold humid Dfa climate to − 01 ± 2.3 Mg C ha^−1^ in a hot arid Bsh climate. However, variability across climatic zones was less well explained by the considered variables, most likely due to similar effects of temperature and moisture change on C input and C mineralisation. Initial SOC was the sole explanatory variable of the best model (R^2^ = 0.43).

**Table 1:** Average absolute soil organic carbon (SOC) stock changes for 1919–2018 (100 years) and 1968–2018 (50 years) for the scenarios NPP_const_ (constant net primary production) and NPP_var_ (variable net primary production) for all climatic zones based on the Köppen–Geiger classification with standard deviation (sd) and number of grid cells (n).

Climatic zone		n	NPP_const_ 100 years		NPP_const_ 50 years		NPP_var_ 100 years		NPP_var_ 50 years	
			mean	sd	mean	sd	mean	sd	mean	sd
Af	Tropical, rainforest	41,451	− 1.5	3.6	− 1.5	2.7	− 1	3.1	− 0.8	2.2
Am	Tropical, monsoon	43,067	− 0.9	2.1	− 0.9	1.9	− 0.7	2.2	− 0.6	1.8
Aw	Tropical, savannah	126,186	− 1.3	2.8	− 0.8	1.6	− 1.3	2.4	− 0.9	1.5
BWh	Arid, desert, hot	108,260	− 0.6	1.2	− 0.2	0.6	− 0.1	2.3	− 0.1	1.3
BWk	Arid, desert, cold	49,457	− 1	0.8	− 0.5	0.4	− 0.5	1.5	− 0.5	0.9
BSh	Arid, steppe, hot	84,275	− 0.6	2.2	− 0.2	1.5	− 0.3	2	− 0.2	1.4
BSk	Arid, steppe, cold	87,719	− 1.5	1.8	− 0.7	1.2	− 1.2	2.1	− 0.9	1.5
Csa	Temperate, dry summer, hot summer	21,739	− 1.8	2	− 1.2	1.4	− 2.8	2.5	− 1.5	2.1
Csb	Temperate, dry summer, warm summer	9029	− 2	3.9	− 0.5	2.5	− 3.7	4.8	− 1.9	3.1
Csc	Temperate, dry summer, cold summer	58	− 2.1	2.9	− 1.1	1.7	− 6.3	5.8	− 3.6	4
Cwa	Temperate, dry winter, hot summer	32,502	− 1.6	2.2	− 0.8	1.4	− 1.4	1.8	− 0.8	1.2
Cwb	Temperate, dry winter, warm summer	9750	− 1.2	4.2	− 0.3	2.6	− 1.6	3.4	− 0.6	2.1
Cwc	Temperate, dry winter, cold summer	60	− 0.9	7.4	0	4.5	− 1.7	5.5	− 0.1	3
Cfa	Temperate, no dry season, hot summer	57,046	− 4.1	4.5	− 2.3	3.4	− 2	3.3	− 1.4	2.5
Cfb	Temperate, no dry season, warm summer	53,051	− 5.6	6	− 4.1	4.8	− 3	4.1	− 2.6	3.6
Cfc	Temperate, no dry season, cold summer	9105	− 10.8	7.2	− 7.3	5.1	− 6.2	5.1	− 4.1	3.7
Dsa	Cold, dry summer, hot summer	3082	− 1.9	1	− 1	0.7	− 2.2	1.6	− 1.4	1.3
Dsb	Cold, dry summer, warm summer	4923	− 2.2	1.6	− 1.2	1	− 1.5	1.5	− 0.8	1.2
Dsc	Cold, dry summer, cold summer	3176	− 4.7	6.5	− 2.5	4.3	− 3	5	− 1.6	3.2
Dwa	Cold, dry winter, hot summer	9600	− 1.2	2.4	− 0.3	1.5	− 0.1	1.8	−0.1	1.2
Dwb	Cold, dry winter, warm summer	3673	− 1.9	3.1	− 1.2	1.9	− 0.6	2.3	− 0.5	1.5
Dwc	Cold, dry winter, cold summer	1828	− 1.2	3.7	− 1.3	1.6	0	3.4	− 0.4	1.4
Dfa	Cold, no dry season, hot summer	25,025	− 9.2	8.6	− 4.6	4.3	− 6.4	7	− 3.5	3.5
Dfb	Cold, no dry season, warm summer	70,581	− 4.7	4.9	− 2.9	3.2	− 2.5	3.7	− 1.7	2.4
Dfc	Cold, no dry season, cold summer	24,164	− 7.4	7.3	− 5	5.4	− 3	4.7	− 2	3.4
Et	Polar, tundra	53,310	− 3.7	6.2	− 3	4	− 2.2	5	− 1.7	3.2
Ef	Polar, frost	26	− 4.2	3.2	− 1.7	0.7	− 5.9	5.1	− 2.7	2.2

**Table 2:** Average relative soil organic carbon (SOC) stock changes for 1919–2018 (100 years) and 1968–2018 (50 years) for the scenarios NPP_const_ (constant net primary production) and NPP_var_ (variable net primary production) for all climatic zones based on the Köppen–Geiger classification with standard deviation (sd) and number of grid cells (n).

Climatic zone		n	NPP_const_ 100 years		NPP_const_ 50 years		NPP_var_ 100 years		NPP_var_ 50 years	
			mean	sd	mean	sd	mean	sd	mean	sd
Af	Tropical, rainforest	41,451	− 2.6	5.7	− 2.8	4.8	− 1.4	4.3	− 1.2	3.1
Am	Tropical, monsoon	43,067	− 2.0	3.7	− 1.7	3.4	− 1.3	3.7	− 0.9	3.2
Aw	Tropical, savannah	126,186	− 2.9	5.9	− 1.8	3.9	− 3.1	5.0	− 2.2	3.4
BWh	Arid, desert, hot	108,260	− 3.1	4.2	− 1.5	3.2	− 0.1	8.0	0.1	5.5
BWk	Arid, desert, cold	49,457	− 3.9	2.0	− 2.0	1.2	− 1.9	4.6	− 2.2	3.2
BSh	Arid, steppe, hot	84,275	− 1.3	7.2	− 0.4	5.6	− 1.0	6.9	− 0.4	4.9
BSk	Arid, steppe, cold	87,719	− 3.8	3.6	− 1.9	2.5	− 2.8	4.4	− 2.2	3.1
Csa	Temperate, dry summer, hot summer	21,739	− 3.7	4.0	− 2.6	2.7	− 5.7	4.1	− 3.1	3.9
Csb	Temperate, dry summer, warm summer	9029	− 2.4	4.5	− 0.6	3.3	− 5.1	5.0	− 2.9	3.8
Csc	Temperate, dry summer, cold summer	58	− 2.0	1.6	− 1.3	1.1	− 8.4	6.2	− 4.9	3.7
Cwa	Temperate, dry winter, hot summer	32,502	− 3.7	4.3	− 2.1	3.1	− 3.3	3.7	− 1.8	2.5
Cwb	Temperate, dry winter, warm summer	9750	− 2.0	7.2	− 0.6	4.4	− 2.9	5.5	− 1.3	3.4
Cwc	Temperate, dry winter, cold summer	60	− 0.4	6.5	0.2	4.3	− 1.7	4.4	0.2	2.7
Cfa	Temperate, no dry season, hot summer	57,046	− 6.6	6.4	− 3.7	4.5	− 3.5	5.6	− 2.4	3.9
Cfb	Temperate, no dry season, warm summer	53,051	− 4.8	4.3	− 3.5	3.4	− 2.7	3.2	− 2.2	2.5
Cfc	Temperate, no dry season, cold summer	9105	− 6.4	3.6	− 4.4	2.6	− 3.8	2.8	− 2.5	2.0
Dsa	Cold, dry summer, hot summer	3082	− 5.7	2.0	− 3.1	1.5	− 6.2	3.2	− 3.7	2.9
Dsb	Cold, dry summer, warm summer	4923	− 5.9	2.3	− 3.1	1.8	− 4.0	2.9	− 2.1	2.5
Dsc	Cold, dry summer, cold summer	3176	− 6.0	3.7	− 3.1	2.8	− 3.8	3.6	− 1.9	2.7
Dwa	Cold, dry winter, hot summer	9600	− 2.7	4.9	− 0.5	3.3	− 0.3	4.0	− 0.1	2.7
Dwb	Cold, dry winter, warm summer	3673	− 3.0	4.9	− 2.0	2.8	− 0.7	4.1	− 0.6	2.6
Dwc	Cold, dry winter, cold summer	1828	− 1.8	4.8	− 2.0	2.2	− 0.1	4.3	− 0.7	1.9
Dfa	Cold, no dry season, hot summer	25,025	− 11.8	7.0	− 6.5	4.2	− 8.3	6.5	− 4.8	3.7
Dfb	Cold, no dry season, warm summer	70,581	− 5.3	4.3	− 3.4	3.1	− 3.0	3.8	− 2.1	2.6
Dfc	Cold, no dry season, cold summer	24,164	− 7.8	4.3	− 5.4	3.7	− 3.3	3.7	− 2.2	2.9
Et	Polar, tundra	53,310	− 4.2	5.3	− 3.2	3.3	− 2.4	4.3	− 1.7	2.5
Ef	Polar, frost	26	− 3.7	1.0	− 1.6	0.4	− 4.8	3.3	− 2.4	1.6

**Figure 3:**
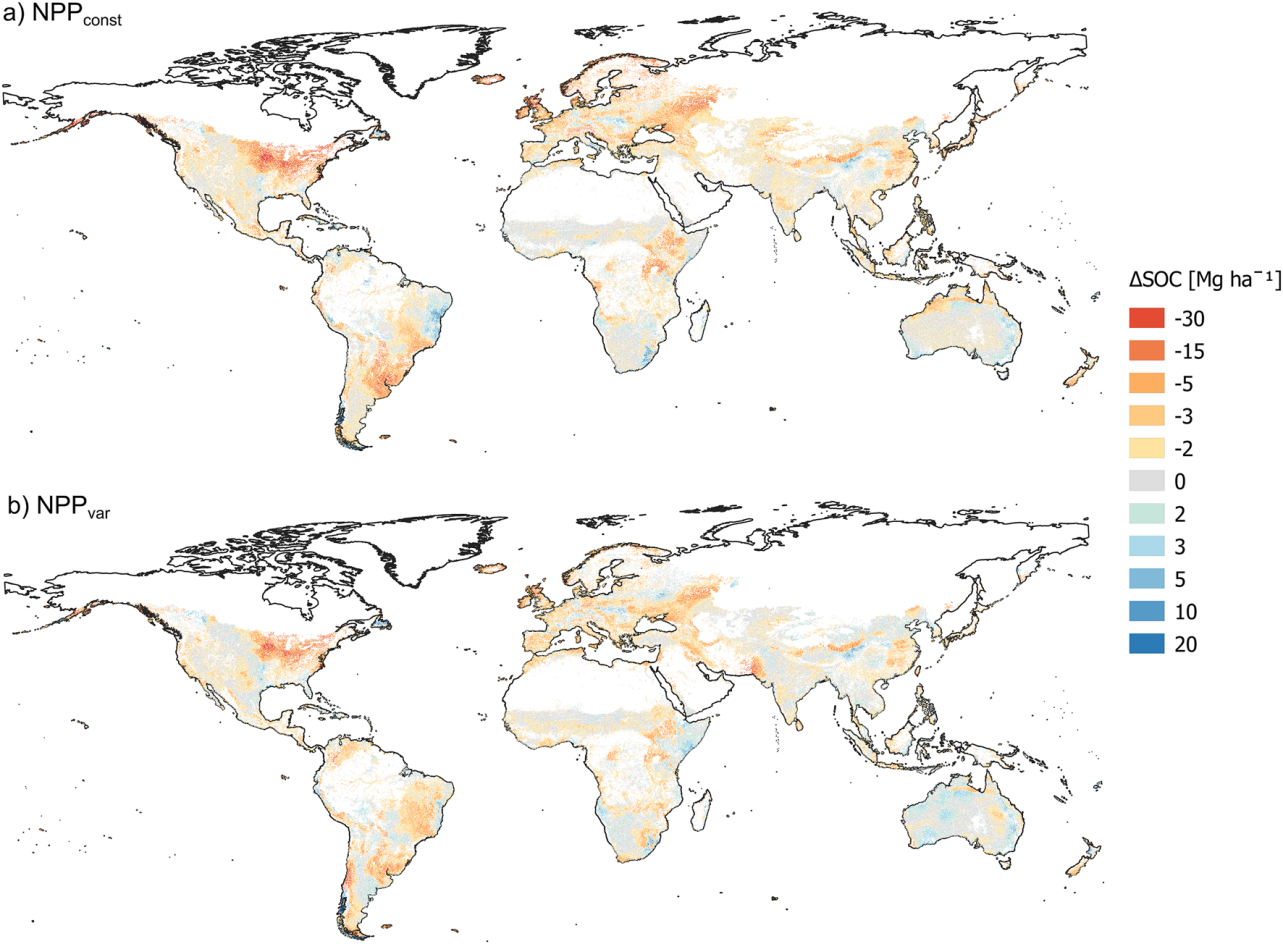
Global distribution of climate change-driven soil organic carbon (SOC) stock changes in agricultural topsoils between 1919 and 2018 for scenarios with (**a**) constant net primary production (NPP_const_) and (**b**) variable net primary production (NPP_var_). White areas are those with non-agricultural land cover. 0 changes (grey) indicate changes of 0±0.3 Mg C ha^−1^ and all other values indicate changes ranging up to the given number. Maps were created in R, version 4.1.1 (https://cran.r-project.org/) using the package *terra*^26^.

**Figure 4:**
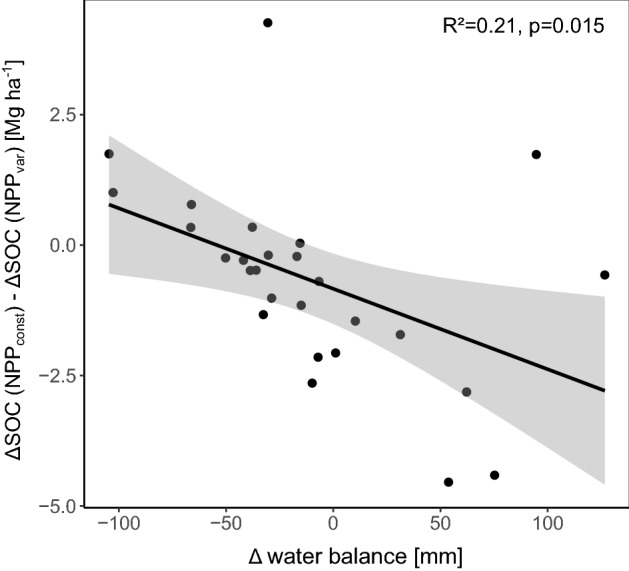
Difference in soil organic carbon (SOC) stock changes between the two model scenarios (constant net primary production, NPP_const_, and variable NPP, NPP_var_) for each climatic zone as a function of the change in water balance (a positive change implies more available water). Linear regression with standard deviation is shown.

When mapped globally, spatial patterns of SOC stock change revealed increases in areas with negative temperature trends (cooling). The most pronounced SOC increases could be seen in south-west Canada, south-east USA and parts of South America, Africa, China and Australia (Fig. 3). However, in some extreme cases, such as in parts of Australia, even the negative trend in precipitation caused the model to predict an increase in SOC, with the decay of unchanged organic matter input becoming water-limited (Fig. S2). This is in line with climatic zones in which the water balance became more negative tending to lose less SOC (Fig. 2). This was not the case for the NPP_var_ scenario, since the soil moisture responses of plants and soil microorganisms have opposing effects on SOC stocks. Therefore, the difference between SOC losses in the NPP_const_ and NPP_var_ scenarios was negatively correlated to changes in water balance (Fig. 4). This indicates that in regions that became drier over the past century, NPP was negatively affected and thus more SOC was lost in NPP_var_ compared with NPP_const_. However, in the majority of climate zones (20 out of 27), increased plant growth partly compensated for SOC losses (Table 1).

**Figure 5:**
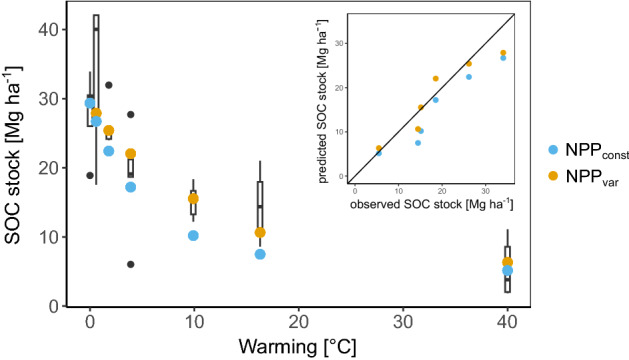
Measured and modelled steady-state soil organic carbon (SOC) stocks along a geothermal warming gradient on an Icelandic grassland soil. Boxplots represent measured data (n = 5) with black dots indicating outliers, while blue and organge dots represent the two modelling scenarios assuming constant C input (NPP_const_) and variable C input (NPP_var_).

A geothermal warming experiment was used to validate the scenarios used in this study. Figure 5 clearly depicts, that RothC, as well as the combination of RothC and MIAMI were able to predict the general trend in SOC stocks with relatively high accuracy. Relative root mean square errors (RMSE) were 26% for NPP_const_ and 19% for NPP_var_. The NPP_var_ scenario was slightly closer to the observed results, which can be explained by the fact that the positive impact of warming on NPP partly compensated for enhanced mineralisation. This plausibility check increases the confidence in the global modelling results.

## Discussion

This is the first time that a comprehensive and spatially explicit estimate is provided on the contribution of climate change to global agricultural SOC stock dynamics over the past century. As expected, the average impact was negative and more pronounced in the latter half of the century, in accordance with the global average temperature trend (IPCC). Over the course of 100 years in which the global air temperature was warmed by on average 1.03 °C, agricultural soils lost on average 1.6–2.5 Mg C ha^−1^ (depending on the scenario) or were relatively depleted by 2.5–3.9%. This is in line with the order of magnitude observed in the very few long-term warming experiments that have been undertaken. In a century-long geothermal soil warming gradient in subarctic Canada, whole-soil SOC stocks have been found to decline by 3% per 1 °C^27^ in a deciduous forest. This was also observed for a grassland topsoil in a geothermal gradient in Iceland (2.8 Mg C ha^−1^ °C)^28^. Finally, the Harvard Forest soil, the longest-running soil warming experiment, lost about 3 Mg C ha^−1^ °C^−1^ in 26 years of warming, which is also similar to the estimated absolute values in the present study^29^. Long-term warming experiments in agricultural systems are scarce, so we used the mentioned Icelandic, geothermal warming experiment on a semi-natural grassland soil to validate the applied modelling approaches. Despite the facts that i) the studied soil was an Andosol which can have distinct properties regarding SOC stabilisation, (ii) warming occurred abruptly, from below and up to extreme temperatures, both modelling approaches were predicting the observations surprisingly well. The NPP_const_ scenario tended to slightly overestimate SOC losses, indicating that NPP and C inputs were also affected by soil warming. The validation exercise increased our confidence in the model results and proves that also relatively simple first-order kinetics models can be applied to estimate climate-change driven SOC stock changes.

With an average absolute change rate of − 0.019 (NPP_var_) and − 0.032 Mg C ha^−1^ year^−1^ (NPP_const_) since 1968, the results of this study suggest that the contribution of climate change to observed trends in SOC stocks is relatively small, yet not negligible. In relative terms, SOC was lost at rates of 0.03–0.05% per year, which is one order of magnitude below the SOC losses (0.6% year^−1^) observed in England and Wales in 1978–2003, for example^10^. However, the values are well in line with a model estimate of the climate change-driven annual SOC loss for the same region (− 0.08, − 0.04 and − 0.05% for cropland, grassland and forest mineral soils respectively without taking NPP changes into account)^12^. Both comparisons, i.e. with the long-term warming experiments (including the validation site) and the regional model exercise, indicate that the results of the present study are realistic. Thus, climate change is having an evidently persistent and extensive negative effect on global SOC stocks, but is about one order of magnitude lower than SOC losses observed in inventories of past decades^10,13,30^.

SOC dynamics are strongly driven by soil temperature and moisture. Both are highly variable on a global scale, on average and in their temporal trends over the last century. As expected, this has resulted in a diverse pattern of estimated changes in SOC stocks. Gains in SOC in the NPP_const_ scenario can be explained by either decreasing temperature or, in some cases, a more negative water balance (declining water availability), both of which can limit SOC turnover^31^. In some areas, the stimulation of NPP slightly overcompensated for increased SOC mineralisation, causing an accumulation of SOC with past climate change. This fits well with the fact that biomass production is expected to increase most in colder climates^32^. The combination of the two scenarios highlights the complexity of spatially explicit prediction of this particular climate-carbon cycle feedback^33^. However, in the modelling framework used here, no average SOC gains per climate zone were predicted for either of the two scenarios, pointing to the fact that increased NPP and thus C input is unlikely to compensate for SOC losses due to stimulated heterotrophic respiration^34^.

This study focused on agricultural mineral soils since they are the main target area for SOC sequestration measures and the area of applicability for the modelling framework^23^. Forest soils, organic soils and soils in other ecosystems such as wetlands and urban areas were not modelled. Although the directions of change might be similar in these systems, it was not possible to derive estimates for the whole land surface. Therefore, no attempt was made to estimate total climate change-driven losses of SOC, which would be a rather incomplete and imprecise estimate, also because the agricultural area has been far from constant in the past century^35^. Furthermore, the RothC model works in a monthly timestep, which flattens out climate extremes that might have more severely affected NPP and microbial activity^36^. However, it is believed that such short-term effects would not have changed the magnitude of change over the course of one century. Increased atmospheric CO_2_ is also a feature of climate change and has a fertilising effect on NPP^37^. Thus, the NPP response was probably slightly underestimated. However, several studies showed that the effect might be rather insignificant^38,39^. Moreover, backwards modelling was undertaken based on a steady-state assumption of SOC stocks to obtain the annual C inputs for the year 2018. This assumption was a rough approximation for the majority of soils, which may have led to an incorrect initial pool distribution. However, it is believed that this is not problematic as century-scale model projections have been shown to be very robust to variations in initial pool distributions^40^. Moreover, the steady-state was a necessary precondition in this study to exclude any legacy effect and clearly focus on climate change as the only driver of SOC dynamics. It should also be highlighted again, that the modelling approach deliberately ignored all changes in agricultural land use and management in order to isolate the climate change effect on SOC stocks from all other effects. Consequently, the modelled values are of a theoretical nature, particularly for the NPP_var_ scenario. The technological achievements in the last century had huge impacts on agricultural NPP^41^. This was not all accounted for in the present study, while the SOC stocks used to initialise the model runs (2018) were most likely more depleted than they would have been if only climate change affected them in the past century. In consequence, since SOC stock changes are a function of initial SOC stocks^42^, the absolute estimates in this study for the past century are slightly lower than they might have been in reality. However, (i) this is not true for reported relative changes and (ii) the advantage of using current SOC stocks is that the absolute estimates of climate change-driven alterations in the past 50 years can be expected to be close to the rates of today and the near future, since global warming has been a linear process since that time. Finally, Bradford et al. mentioned that many biogeochemical models, which are most often based on first-order kinetics, partly represent outdated knowledge of C turnover^5^. They argue that only the realisation of new ideas about C cycling in soils can increase confidence in modelling results regarding the effects of global warming. One specific example is the physiological response of microbes to warming, which may or may not decrease their growth efficiency^2,43–46^ and thus may or may not affect the size of the microbial biomass pool and also SOC stabilisation and mineralisation^47^. Indeed, experimental results on such specific mechanisms tend to scatter in all possible directions, as do ensembles of more complex biogeochemical models when applied to data of experimental warming^48^. Bradford et al. estimated, that a timespan of about 25 years would be needed to reduce current uncertainties and build more confidence on the overall effect of global warming on SOC stocks^5^. Currently, we see the use of a globally applied first-order kinetics model as a conservative and relatively robust way for such a global modelling effort with results being comparable to the multiple contexts in which RothC or similar models are still state of the art (such as national greenhouse gas inventories). We consider it unlikely, that the steady increase in temperature of on average 1 °C strongly altered microbial physiology and SOC stabilisation mechanisms^2,44^ and also tested the approach on an experimental dataset that was predicted surprisingly well.

Despite several shortcomings and uncertainties, this study provides a robust and spatially highly resolved estimate of climate change-driven SOC dynamics over the past century. This is an important product that may help interpreting observed local SOC changes and provide a certain reference scenario in various contexts. One example could be carbon farming, where currently the discussion is much about the quantification and verification of SOC sequestration on a certain land area^49^. In fact, not all measures implemented will lead to an absolute increase in SOC stocks, but rather avoid SOC losses. Without a seriously estimated reference scenario, it cannot be quantified how much CO_2_ loss due to climate change was effectively avoided. This is taken up by carbon farming start-ups, seeing a chance to sell certificates even after detecting no changes in SOC over time (https://co2-land.org/?page_id=60). The danger of a too negative reference scenario is high. A similar problem exists in forests, in which the baseline scenario (how much of the area would potentially be deforested) determines how much CO_2_ certificates can be sold when the forest is protected. The presented comprehensive estimation of global SOC stock changes attributable to climate change as an omnipresent global phenomenon can serve as a calibration baseline, while it is recommended to use more exact local data and modelling approaches to estimate climate change and possibly other impacts on SOC trends in carbon certification schemes.

## Conclusions

The last one hundred years have seen rapid transformation. Technological developments, such as synthetic N fixation, as well as a rapidly growing human population have paved the way for the onset of large-scale industrial agriculture as well as for ever-increasing CO_2_ emissions responsible for the greenhouse effect^50^. The latter has led to the most severe environmental crisis humanity has ever faced: the climate crisis. Both agricultural intensification and climate change have simultaneously affected agricultural SOC stocks to varying extents, leading to a significant feedback to atmospheric CO_2_. Despite the tremendous efforts being made to quantify changes in SOC stocks in almost all regions of the world, drivers for such changes often remain elusive and, until now, were a blind spot in national greenhouse gas inventories^51^. The contribution of climate change is often subject to speculation and either ignored or inflated. It is ignored when emission factors rather than dynamic models are used to estimate SOC stock change due to land-use or management change^52^, and potentially inflated when changes in observed SOC stocks are explained^10^. The global estimates presented here provide a reliable foundation for a variety of applications into which the persistent, climate change-driven baseline trend of SOC stocks can be integrated.

## Materials and methods

The RothC^24^ model was applied to predict climate change-driven SOC changes on global agricultural soils using the RothC version implementation in the R package *soilassessment*^53^. It is an established first-order kinetics model with five C pools that has been successfully applied across multiple climatic zones and also in climate change projections^54–56^. To estimate only the climate change impact on SOC stocks of today’s global agricultural soils, we had to start with a steady state situation excluding any potential legacy effects on SOC trends. Global SOC stocks are only available in sufficient quality and coverage for recent years, and also the agricultural area of today is not comparable to the agricultural area 100 years ago. Therefore, we initialised the model with todays’ SOC stocks and used temperature and precipitation data of the past 100 years to model SOC development, as affected by climate change only, backwards. In this way, all major agricultural developments in the past century, as well as land use changes were explicitly excluded from the modelling exercise and the model only predicted what a “cooling” effect (warming backwards) might have on SOC stocks. The GSOCmap (FAO-ITPS, 2019, 1 km resolution) was used to specify the initial value of the soil carbon stocks for the 932 k modelled points. In addition to carbon stocks, model driving data were used that are similar to the data compilation suggested by the FAO initiative “*Global soil organic carbon sequestration potential*”^23^:

- clay contents in the 0–30 cm topsoil layer taken from soil texture maps of the OpenLandMap with a 1 × 1 km resolution (https://doi.org/10.5281/zenodo.1476854)
- monthly precipitation, mean temperature and potential evapotranspiration from the CRU TS v 4.03 raster dataset with a resolution of 0.5°^40^
- land use of 2018 from the European Space Agency (ESA) Climate Change Initiative (CCI) – Copernicus Climate Change Service in a resolution of 300 m
- monthly land cover derived by NVDI from MODIS–MOD13A2 datasets (https://lpdaac.usgs.gov/products/mod13a2v006/), after the method suggested in^23^

Prior to model runs, the model was initialised by spin-up runs to derive carbon input at equilibrium (Cin_equi_) and related pool distributions in 2018. These spin-up runs were done with an analytical solution of RothC^42^ to minimise computational time.

**Figure 6:**
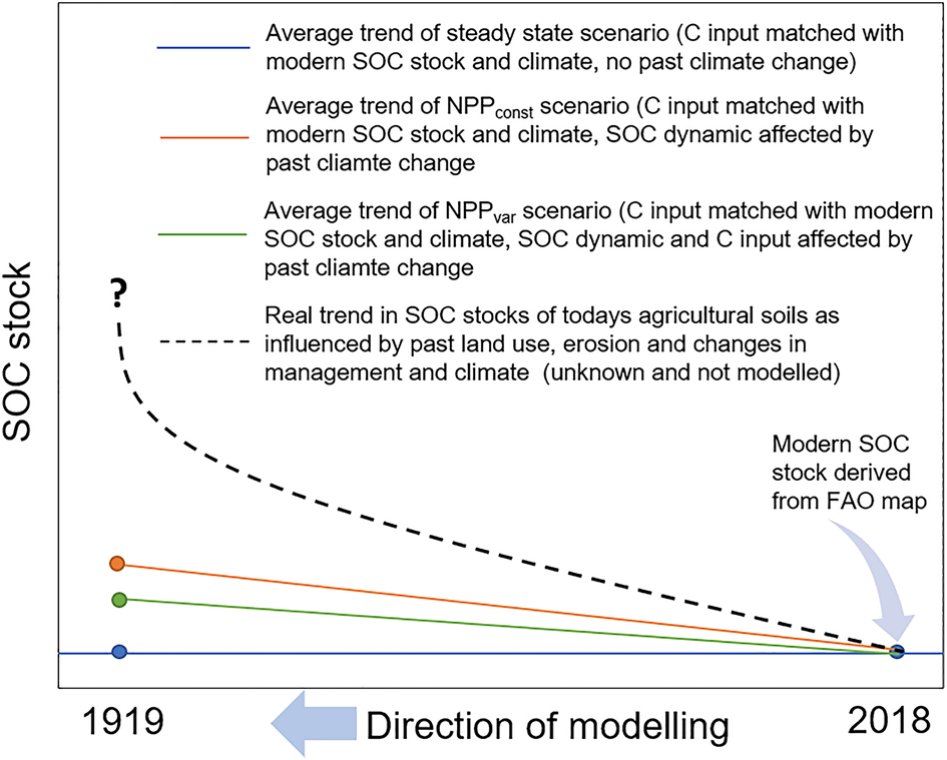
Illustration of the modelling framework with exemplary average soil organic carbon (SOC) stock trends of the two scenarios NPP_const_ and NPP_var_ as well as the actual, yet unknown trend in SOC stocks of today’s agricultural soils.

Two model scenarios were run, and both explicitly ignored any changes in agricultural practices on both SOC decomposition and C inputs. Instead, only potential climate change-driven alterations in SOC were modelled (Fig. 6). In scenario 1 (NPP_const_), a constant annual carbon input similar to the input at equilibrium was assumed. In scenario 2 (NPP_var_), the annual carbon input from 2018 to 1919 was derived by scaling the Cin_equi_ using the ratio of the recent NPP (NPP(t)) and NPP in the reference period 1919–2018 (NPP_ref_):

$$Cin \left(t\right)=Ci{n}_{equi}\times \frac{NPP \left(t\right)}{NP{P}_{ref}}$$

This is not exactly the same approach applied in^23^, however both approaches produce identical C-input estimates. NPP needed to scale C inputs for the RothC model was estimated by the simple and well established MIAMI model^57^ based on annual precipitation (P) and annual mean temperature (T):

$$NP{P}_{T}=\frac{3000}{1+exp\left(1.315-0.0119T\right)}$$

$${NPP}_{P}=3000\left(1-exp\left(-0.0000664P\right)\right)$$

$$NPP=min\left(NP{P}_{T}, NP{P}_{p}\right)$$

Monthly soil water deficit, required to derive the rate modifying factor b^24^ in the RothC model, was quantified in forward mode starting in January 1919. There are several reasons, why those two scenarios were modelled. First of all, there might be different definitions of what is the baseline of climate change-driven SOC dynamics. One could be interested on the accelerated decomposition alone, which might then lead to considerations of how much more C input would be required to (over)compensate such a loss^6^. However, the question could also be, how climate change might have potentially affected the total balance of C input to the soil and SOC mineralisation. This is particularly interesting, when considering the spatial resolution of such alterations in both fluxes. There might be regions in which climate change has actually led to increased SOC stocks due to an overcompensation of enhanced SOC mineralisation by higher photosynthetic activity, while in other regions e.g. increasing drought led to a decline in potential C input. Figure 6 illustrates the modelling approach.

A unique geothermal warming experiment on Iceland was used to validate the model scenarios applied in this study. For lack of a long-term warming experiment in an agricultural system, we used this semi-natural grassland site, on which five transects along strong soil warming gradients were sampled and analysed for SOC contents^58^. The site is located close to the village of Hveragerdi (64° 00′ 0ʺ N, 21° 11′ 09ʺ W) on an Andosol with the topsoil (0–10 cm) having a loamy texture (8% clay, 61% silt and 31% sand), a pH in water of 5.7 and an average bulk density of 0.6 g cm^3^^58,59^. At the time of sampling, soils were warmed for only six years, by on average 0.6, 1.8, 3.9, 9.9, 16.3 and 40 °C, which was due to an abrupt shift in the location of geothermal channels^59^. However, a later resampling on several of these warming levels plus a sampling of a neighbouring older warming experiment showed that six years of strong and abrupt soil warming was enough to reach a new steady state^28^. Therefore, the RothC model was initialised with the SOC stock of the unwarmed reference plots and run into a new steady state for each of the warming intensities adding the average temperature increase to the sampled weather data from 1998 to 2018, while the SOC stock of the unwarmed reference soil and mentioned climate data was used to estimate steady-state C input. This was done for both scenarios (NPPconst and NPPvar) in the exact same way as described earlier.

Owing to computational limitations, scenario runs were done for a sample of 1% of all raster grids selected by random sampling, which resulted in about 932,389 runs given the 1 km × 1 km resolution of the underlying SOC map. Modelled SOC, carbon input, NPP, weather data and soil moisture deficit were stored for 10-year time intervals. These variables were aggregated according to the Köppen–Geiger climate classification map^60^.

Raster maps of modelled soil carbon changes were spatially aggregated to a 0.1° resolution using the *terra* package in R^61^.

Linear regression models were fitted to explain the variability of SOC stock changes (absolute and relative) across climatic zones. Initial SOC stocks, mean annual temperature (MAT), mean annual precipitation (MAP), water balance as well as changes in temperature, precipitation and water balance were used as explanatory variables. The best model was chosen based on the Akaike Information Criterion (AIC) and model residuals were visually checked for normal distribution using quantile–quantile plots.

## Supplementary Information

Supplementary Information.

## Data Availability

The datasets on which this analysis was based is freely downloadable and sources are given in the manuscript. Modelling results are made available at https://doi.org/10.5281/zenodo.7781245.
