# Supplementary material for: The legacy of one hundred years of climate change for organic carbon stocks in global agricultural topsoils
Source: Sci Rep. 2023 May 9;13:7483. doi: 10.1038/s41598-023-34753-0 (PMC10170085; doi:10.1038/s41598-023-34753-0)
Supplement: Supplementary file 1 — Supplementary Information. [file 41598_2023_34753_MOESM1_ESM.docx]

**Supplement**


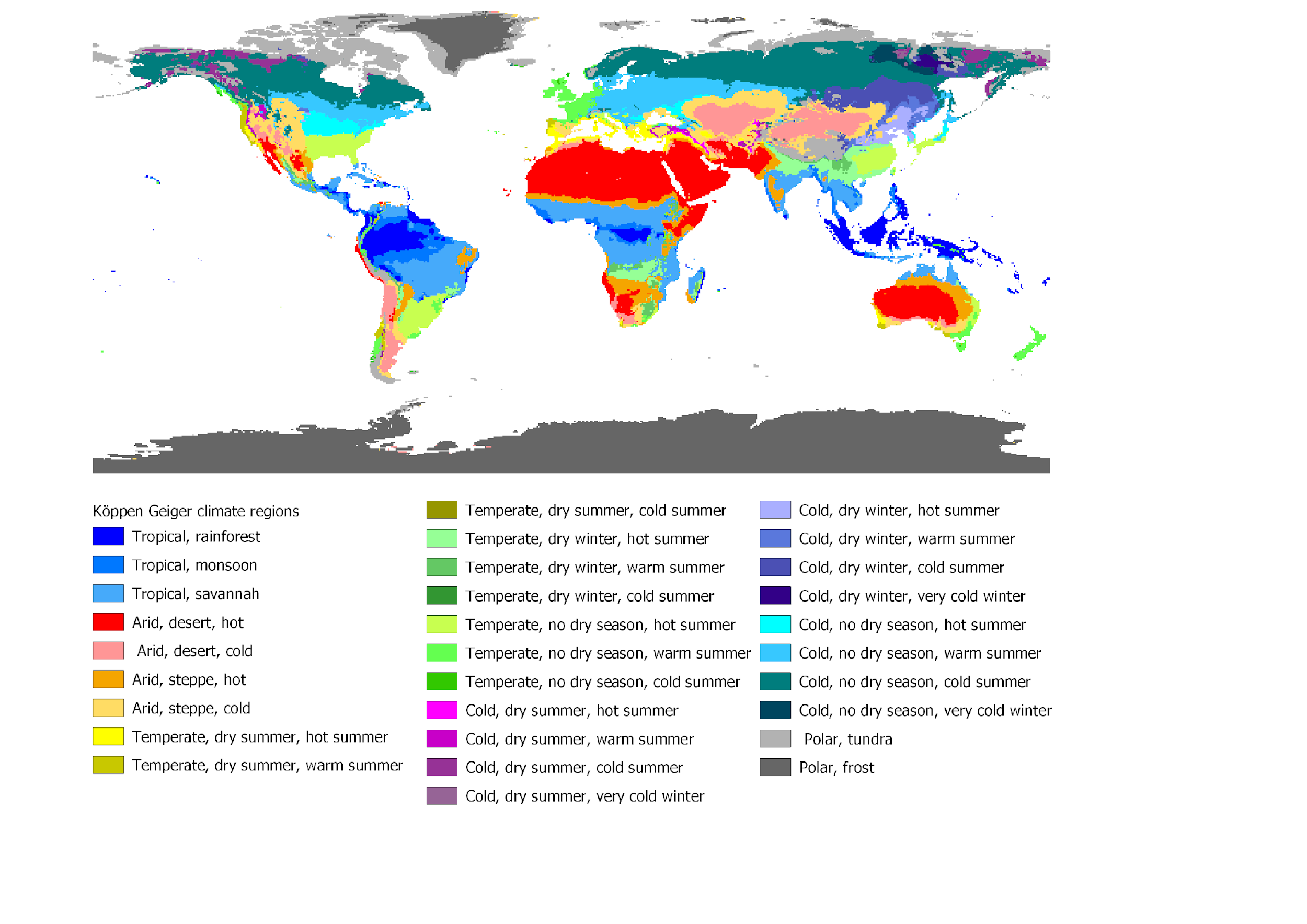


Figure S1: Map of the distribution of climatic zones based on the Köppen-Geiger classification for the period 1980-2016 (adapted from ^45^). Data, which was published under the CC4.0 license (<https://creativecommons.org/licenses/by/4.0/>), was downloaded as GeoTiff from <http://www.gloh2o.org/koppen/> and plotted in QGIS 3.1.6.



Figure S2: Distribution of changes in a) temperature, b) precipitation and c) water balance between 1901-1920 and the reference period 2001-2020. Maps were created in R, version 4.1.1 (<https://cran.r-project.org/>) using the package *terra* ^26^.
